# 

**Published:** 1939

**Authors:** 


Vol. LVI. No. 213 AUTUMN, 1939.

### The Bristol Medico-Chirurgical Journal

PUBLISHED UNDER THE AUSPICES OF

THE BRISTOL MEDICO-CHIRURGICAL SOCIETY.

*Editor:* J. A. NIXON, C.M.G., M.D., F.R.C.P.

WITH WHOM ARE ASSOCIATED

E. W. HEY GROVES, D.Sc., M.S., F.R.C.S.

A. E. ILES, O.B.E., M.B., B.S., F.R.C.S.

A. RENDLE SHORT, M.D., B.S., B.Sc., F.R.C.S.

W. MELVILLE CAPPER, M.B., B.S., F.R.C.S.

E. WATSON-WILLIAMS, M.C., Ch.M., F.R.C.S., *Assistant Editor.*

BRISTOL: J. W. ARROWSMITH LTD., QUAY ST. & SMALL ST.

LONDON: J. W. ARROWSMITH (LONDON) LTD.

FULWOOD HOUSE, FULWOOD PLACE, HIGH HOLBORN, W.C. 1

**Price Three Shillings net.**

